# From bench to clinic and back: Perspective on the 1^st ^IQPC Translational Research conference

**DOI:** 10.1186/1479-5876-2-44

**Published:** 2004-12-20

**Authors:** Heidi Hörig, William Pullman

**Affiliations:** 1 Columbia University Medical Center, Division of Surgical Science NY 10032 USA; 2 Sanofi-Aventis, Bridgewater NJ 08807 USA

## Abstract

Translational Research (TR) provides a set of tools and communication context for scientists and clinicians to optimize the drug discovery and development process. In the proceedings of a Princeton conference on this timely topic, the strengths and needs of this developing field were debated. Outcomes and key points from these discussions are summarized in this article which covers the topics of defining what we mean by translational research (both theoretically and in operational terms), ways in which to engender the TR mindset and embed it in organizations such as the pharmaceutical industry in order to optimize the impact of available technologies (including imaging methods), the scientific basis and under-pinnings of TR including genomics knowledge, information sharing, as well as examples of application to drug discovery and development. Importantly, it should be noted that collaborations and communications between the stakeholders in this field, namely academia, industry and regulatory authorities, must be strengthened in order for the promise of TR to be delivered as better therapies to patients.

## Introduction

There are many challenges facing pharmaceutical companies in the post-genome era not least of which is declining productivity and innovation. Not surprisingly, there is agreement between Industry, Academia and Regulatory communities that the drug discovery and development process needs to change in order to meet the future needs of patients with effective and desirable drugs. A key part of the strategic solution is to leverage the application of TR principles and practices, which if implemented will go a long way towards addressing the challenge posed by FDA's Critical Path Initiative [1] (for more detail on this initiative see section on "Optimizing the Impact of TR" and ). Successful drug development requires satisfying a matrix of domains from relevance to the disease and the drug-ability of the target through feasibility and convenience of drug delivery, demonstration of favorable benefit-risk profile in order to achieve to a drug label that reflects physician and patient acceptance. Herein lies a key role for TR in helping to navigate this journey.

In order to promote discussion on this topic, the International Quality and Productivity Center (IQPC) organized a *Translational Research *Conference (20–22 September 2004 Princeton, NJ) that hosted a small group of clinical and basic science researchers and individuals from the pharmaceutical industry. Among the topics discussed were how to define "Translational Research", how to expedite the transfer of pre-clinical findings to influence development plans, how to select biomarkers to ensure support for decisions, how new strategies can be effectively translated to practical tactics, and what team players and collaborations are necessary to conduct successful TR. Success factors identified include: Identification and validation of novel drug targets, development of robust and validated assays to screen drug leads for safety and potential efficacy in humans, and the identification of suitable patients for expedited but informative trials.

## Defining Translational Research

While the goal of TR is to implement *in vivo *measurements and leverage preclinical models that more accurately predict drug effects in humans, TR itself can be defined in many ways. At its core however, is the thesis that information gathered in animal studies can be translated into clinical relevance and vice versa, thus providing a conceptual basis for developing better drugs. It could in fact be argued that the designation of a special term or definition for TR might be unnecessary or even misleading. Historically the term was assigned to create awareness and advocacy for the general public, clinicians and scientific communities, and especially for the government and other private sponsors [2] in this evolving discipline. Nonetheless, the basis for TR lies in sound scientific and clinical research principles.

Whatever the precise definition TR, it should serve as a forum to find a "common language" for clinicians and scientist in navigating the complexities of basic scientific approaches, data analysis and information processing. It clearly implies the need for an intensive training for scientists and clinicians in multiple disciplines to acquire expertise and experience to conduct TR. For the purposes of this symposium, the scope of translational research was defined as the application of scientific tools and methods to drug discovery and development. This can be achieved by integrating information concerning a) exposure (pharmacokinetics), b) biological activity (pharmacodynamics including safety profiles) delineating differences between species and leading to the validation of target and mechanism biomarkers, and c) outcomes leading to an understanding of efficacy and safety between species and ultimately to the qualification or linkage of biomarkers to clinical outcome (for a fuller discussion on Biomarkers and Surrogate Endpoints see definitions in [3]). Thus TR can be used to mitigate risk and enhance drug development opportunities.

Therefore, in taking a pragmatic or operational rather than a definitional approach, a key to a successful translation of non-human research to human clinical trials lies in the choice of biomarkers. While biological pathways tend to be homologous across species and more so than pharmacokinetic parameters such as absorption and clearance, animal models themselves have a poor record of predicting human disease outcome. Nonetheless, biomarkers are the key for prediction of biological activity if not drug efficacy in humans. At least three types of biomarkers can be identified: (1) target biomarkers measuring the interactions between a drug and its target; (2) mechanism biomarkers measuring their downstream biological effects and (3) outcome biomarkers that reflect efficacy and safety. A second dimension can also be ascribed to biomarkers to help drug developers assign risk assessment to such approaches. This sub-classification links desired utility to points on a risk continuum; e.g. low, medium and high, in which 'low' describes a biomarker applied solely to animal models for example for selecting compounds for progression into humans, whereas 'medium' is association with utility for some aspects of early clinical profiling of efficacy and safety including across species correlation, and 'high' is associated with reproducibility and qualification as an outcome or even regulatory tool in humans.

Additionally, TR itself undergoes an evolution from pathfinding (hypothesis generating) to discovery research, to development, and finally to application. Each of these operational phases is amenable to being evaluated or supported by biomarkers, either for the definition of objectives, proof of principle or in assessing risk and feasibility. Consequently the right choice of biomarkers can help drive decision-making and lower the costs and cycle-time for progression of a new drug from the bench into the clinic. In summary, whatever the definition or classification ultimately used, in practical terms translational tools should be developed and applied on a "fit for purpose" basis with prior assessment and agreement of attendant risks.

## Optimizing the Impact of Translational Research

Traditional Research and Development (R&D, also referred to as Discovery and Development) paradigms have accentuated the boundaries between the territories of discovery and development worlds and have not been conducive to bridging key transition points. This is unfortunate since the development world tends to lag behind advances made in discovery, a point recognized by FDA in launching the Critical Path Initiative [1]. In brief, this initiative challenges Industry and others to develop and implement better tools, such as biomarkers, trial modeling and simulation and other solutions, in order to optimize the development and regulatory stages of a product's life.

While advances have been made on streamlining forward progression of R&D through organizational linkages, what has not happened to the same degree is a bi-directional flow of information, namely flow of information from the clinic (e.g. clinical validation or lack thereof) back into the hands of the discovery scientist. The consequence of this is that the biological models used to qualify drug candidates may fail to be predictive of subsequent drug responses in the clinical setting. Thus a practical outcome of TR is to improve the overall probability for technical success (POS) in drug development.

Consequently the next paradigm for R&D optimization depends not only on leveraging emerging technologies such as pathway mapping and *in silico *modeling, but also the need to empower key scientists and clinicians with the task of enhancing the prediction and iteration learning cycle. Since there are different organizational solutions for embedding the TR mindset within an organization, a key element is to provide TR expertise to drug development teams. Furthermore, innovation and productivity values are critically linked through information exchange. Rapid iteration (e.g., learn-confirm cycles) and transfer of knowledge gained from prototype development experience will enable more rapid compound redesign against the highly desired target and be reflected as enhanced innovation. On the productivity side, the tools outlined in the Critical Path Initiative [1], once effectively implemented, will lead to enhanced development productivity but only if information exchange occurs efficiently across different functions. Hence a backbone for TR is support by user-friendly informatics systems.

The journey however starts at understanding the scientific foundations of physiology and pathophysiology, thus providing a rational linkage between the gene, its expressed product, disease expression and ultimately outcome. The discipline of biomarker identification and development as mentioned previously encompasses these principles and is a core tool in the TR scientist's armamentarium.

Biomarkers (which are not necessarily Surrogate endpoints and few are in fact) are key tools for escorting the drug candidate from the bench to the bedside and back. That is they can be both animal "diagnostic" as well as human "diagnostic" tools. A key implementation tool is therefore to identify early on which biomarkers may be of value and to study these in the relevant animal models, that is, specifically include them in preclinical screening paradigms, as well as identify their role (e.g., go / no go decision factors) in the clinical development plan. Biomarkers, which include imaging techniques as well as protein and genetic markers, may fulfill several roles in R&D from compound screening and selection through dose justification, decision-making and risk mitigation, however the key is to overtly link them to the discovery and development plans with *a priori *agreed performance characteristics, such that there is agreement on the utility of the marker.

There are many good examples of the value or non-value of preclinical models in predicting subsequent human response and safety. The journey from preclinical experience to the clinic is a well-worn one (e.g., Xenograft testing for oncology), albeit without the degree of overall predictiveness we would desire. On the other hand there is a marked paucity of examples in which clinical experience or observation was translated back into a legitimate drug target and discovery effort (e.g. Viagra). Thus, a major opportunity lies in both developing more sensitive and specific animal models of disease (e.g. knock in/out) as well as fully leveraging novel clinical observations. At the same time it is the ultimate validation in the clinic that counts, and rapid feedback of that information will allow the conditional probabilities and learning cycle to be enhanced. By enabling these principles through organizational and cultural change, the impact of TR will be determined by direct impact on high-quality mid-phase transitions as well as reduced cycle-times and resource burdens.

## Basic science, genomics and Translational Research

The era of genome-scale biology has seen an increase in, and production of, vast amounts of biological data together with an extensive increase in biology-oriented databases. To make the best use of biological databases and the knowledge they contain, different kinds of information from different sources must be integrated in ways that make sense to biologists. A major component of the integration effort is the development and use of annotation standards such as ontologies. Ontologies offer a conceptualization of domains of knowledge and facilitate both communication between researchers and the use of domain knowledge by computers for multiple purposes. Therefore, the Gene Ontology (GO) project was founded in 1998, in an attempt to provide consistent descriptors for gene products, in different databases; and to standardize classifications for sequences and sequence features. Since then, the GO Consortium has grown to include many databases, including several of the world's major repositories for plant, animal and microbial genomes [4]. Despite vast differences in genome size among various species, genes can be highly conserved at the level of protein sequence allowing searching for an unknown human protein function in yeast. As new genome sequences are being rapidly generated, and where comparative genome analysis requires the integration of data from multiple sources, it is especially relevant to provide rigorous ontologies that can be shared by the scientific community at large.

In the past, biological processes and the underlying genes, proteins, other molecules and environmental factors, have been studied separately more than on an integrated basis. The challenge, however, for future research on human disease is to understand not only the mechanistic basis, but also the underlying dynamics of gene product expression. Thus, biological research should emphasize the analysis of pattern of gene expression over individual measurements.

GO has been developed to predict behavior of entire biological systems, being assigned to three aspects: (1) *Molecular Function *describes activities, such as catalytic or binding activities, at the molecular level, e.g. kinase activity. (2) *Biological Process *describes biological goals accomplished by one or more ordered assemblies of molecular functions, e.g. 'cell death' can have both subtypes, such as 'apoptosis', and subprocesses, such as 'apoptotic chromosome condensation'. (3) *Cellular Component *describes locations, at the levels of subcellular structures and macromolecular complexes, e.g. 'nuclear inner membrane' with the synonym 'inner envelope' [4].

The powerful use of comparative gene expression analysis in human disease was exemplified with a recent study on gene expression profiles of gastric cancer patients and their correlation to survival. Leung et al. [5] have shown that Phospholipase A2 group IIA (PLA2G2A) expression is associated with prolonged survival and less frequent metastasis by studying gene expression patterns in human gastric cancers. This observation was confirmed in an independent set of patient samples by using quantitative RT-PCR. Beyond its potential diagnostic and prognostic significance, this result suggested that the activity of PLA2G2A may suppress progression or metastasis of human gastric cancer.

In summary, the application of mathematical models and computer simulations to analyze gene expression profiles and to compare complex data sets of various origins may provide new insight into the pathogenesis of cancer progression and metastasis. The Gene Ontology (GO) project  provides structured, controlled vocabularies and classifications that cover several domains of molecular and cellular biology and are freely available for community use in the annotation of genes, gene products and sequences.

## Translational Research in Drug Discovery: Strategies for Complex Systems

Cancer vaccines are promising therapeutics designed to elicit immune responses against antigens expressed by tumor cells. However, vaccines that have worked well in preclinical models have not translated into consistent responses in the clinic. Since vaccines are comprised of multiple components, multiple immunological endpoints are used to identify the least effective vaccine components in cancer patients. Post-clinical research strategies are subsequently designed with a focus on improving the least effective vaccine components.

To improve the performance of cancer vaccines in the clinics, which are traditionally judged by clinical endpoints, novel endpoints and biomarkers are needed to assist in understanding why cancer vaccines are not working. From clinic to bench, a systematic strategy is needed for pre-clinical optimization that addresses vaccine limitations identified in the clinics; and from bench to clinic, performance criteria need to be established for a follow-up clinical study. After gathering the therapeutic options, testing has to be prioritized on the basis of: a) already available data; b) availability of the therapeutic modality; c) models and assays available internally; d) turnaround time; and e) on the patent landscape.

Prioritization and rapid evaluation of novel therapeutics will decrease the turnaround time and facilitate decision-making. However, several tools are needed to make this a reality. For example, complex therapeutic strategies require biomarker or even surrogate endpoints from clinical trials to direct development of second-generation therapeutics. The rapid qualification and choice of surrogate endpoints should be based on knowledge gathered by an "early-stage therapeutic opportunities database". This comprehensive database should include data on therapeutic targets, models, assays and published results and indeed the plethora of new therapeutic strategies in preclinical stages can only be managed by accessing informative databases. Moreover, pre-clinical compound optimization can be facilitated by establishing quantitative endpoints of short duration and lastly go / no go decision points must be established for surrogate endpoints and clinical responses in animal models.

However, several current issues of scientific basis also have to be addressed, such as the importance of clinical surrogate endpoints, the relevance of animal models, lack of concordances between assays, and the lack of concordance between surrogate endpoints and the clinical response, in order to improve cancer vaccine development strategies.

## Applying Translational Research to Drug Development

A core principle of TR revolves around validation of targets, biomarkers and treatment modalities in humans. These activities and drug development itself cannot be undertaken without patients or clinical data. How TR can be integrated in a multi-center, multi-cultural organization involving patient accrual from more than 38 different countries worldwide, for the research and treatment of cancer can be exemplified by EORTC , a non-profit organization conducting more than 100 clinical trials and treating 7000 cancer patients yearly.

Advancement in basic science and immunology and an overwhelming revolution of biotechnology have changed the targets and endpoints in cancer trials from the mere assessment of cytotoxicity to defined mechanisms for potential anti-tumor effect. That is in the era of "targeted therapies" molecular therapeutics are now being designed to target "strategic" checkpoints that underlie the malignant phenotype. The challenges to be met are: 1) dealing with new compounds affecting novel molecular targets, 2) innovation in design and analysis of clinical trials, 3) cooperation between translational researchers and network of clinical investigators and 4) informed patients. The major concerns in conducting clinical trials are rising costs coupled with efficacy rates as low as 5% in cancer patients, making signal to noise detection not only difficult but expensive.

The need for research on tumor tissue requires the set-up of tumor banks and the associated administrative burden often discourages young oncologists. EORTC established a tumor bank comprising real tissue samples but including a "virtual review" by pathologists. This ensures the availability of a well-categorized and prognostically evaluated collection of primary tumors and allows an online-searchable bank for researchers to access. Indeed the tumor bank harbors paraffin-embedded tumors, as well as frozen tumor tissues  and storage of tissue is de-centralized at the institute where it is collected. To assure equal quality of tissue, which in outcome of scientific experiments can be compared, standardization of the collection and storage methods is fundamental. Therefore, protocols for storage, retrieval and tracking of tissues will be standardized and implemented in all participating laboratories.

Access to the tumor bank allows screening of many available tumor samples for the expression of molecular targets and will help to unravel novel biomarkers for diagnosis and treatment. Such access will allow us to overcome the missed opportunities due to lack of tissue collection in clinical trials, which could have allowed better pre-screening of potentially responsive patients based on expression of certain biomarkers e.g., expression of bcl-2, and the treatment of target positive patients may have ensured a better clinical outcome in this target class.

The challenge of testing promising new modalities for the cure of disease that had shown efficacy in experimental models lies in a lack of understanding of the underlying mechanism, heterogeneity of human genetic backgrounds and a lack of suitable controls in human studies. Strategies have been developed at the NIH for the global monitoring of patients by studying, with high-throughput technology, the systemic effects of treatments as well as their effect within the target organ. For this bedside to bench effort, a systematic sampling of human tissues of local (site of immunogen application), systemic (circulation) and peripheral (tumor site) origin needs to be standardized to ensure high quality of samples avoiding degradation of protein, RNA and DNA. This TR approach allows experimental studies in human samples during or after therapy through amplification of transcripts for analysis of minimal sample tissue, and the application of monitoring techniques for genetic profiling. Further, proteomic-based approaches allow following the kinetics of the mechanism of actions of therapeutics.

Studying the effects of treatment in a bedside to bench approach provides markers for the characterization of disease process and/or testing hypotheses generated by experimental models. Therefore, the nature of research in the clinical setting can realistically be described as 'hypothesis generating", rather than 'hypothesis driven', through a discovery-driven approach. Analysis of the genetic background can reveal polymorphism of genes involved in immune reactions, such as cytokines and their receptors, which might influence the outcome of immunological interventions in different patient populations [6].

Analysis of disease heterogeneity can be approached by transcriptional analysis, through linear amplification of RNA and subsequent analysis by cDNA array and transcriptome array, and/or functional protein analysis, through protein characterization by proteomics [2,7]. Numerous tumor-antigen based cancer vaccine studies have shown that there is a functional dissociation between systemic circulating cytotoxic T cells and tumor infiltrating T cells (TIL). Tumor antigen-specific T cells have been demonstrated to have a quiescent phenotype and consequently cell cycle activation requires antigen-specific stimulation, as well as non-specific stimulation by IL-2 [8]. In addition, the local release of immune inhibitory factors by tumor cells is influencing the T cell phenotype and cytotoxicity leading either to tumor regression or recurrence [2]. To understand these complex mechanisms, it is important to study the tumor microenvironment by collection of large libraries of relevant clinical specimen, such as excisional biopsies or fine needle aspirates (FNA). FNA have the advantage to allow serial sampling of the same tumor site over time and treatment and to allow a prospective follow up of a given lesion. Studying of the tumor microenvironment will provide invaluable insights into mechanisms involved in disease progression and/or changes affected by therapy, in terms of genes whose expression changed due to (1) genetic instability, (2) immune selection or (3) immune regulation.

Despite the many obstacles in monitoring therapeutic effect in early phase clinical trials and the lack of hypothesis, the scientific significance of these trials should be reviewed assuming that the new treatment will not be beneficial. Desirable outcomes include learning about the disease process, the primary goal of the therapy and the reasons for its failure. Another concern should be if we have taken advantage of the patient population accrued at least to learn something, although independent of treatment, about the disease process itself. Clinical trials should therefore be designed, within ethical constructs, to look at questions beyond the ones related solely to treatment. This can be achieved through (1) establishment of libraries of relevant clinical samples for immediate or future studies, (2) prospective collection of data into a consistent format, and (3) tight link between clinical and scientific data.

Developing better therapies for chronic inflammatory diseases also exemplifies the use of the latest technological advances in TR such as proteomics, transcriptomics and cellomics, for identification or application of biomarkers. Chronic inflammation frequently precedes the development of cancer in adults, such as lung [9], esophageal, gastric and pancreatic cancers. This may be due to a switch from apoptotic (scheduled) to necrotic (unscheduled) tumor cell death induced by mechanisms related to the chronicity of the inflammatory response. Acute inflammatory processes caused by viral or bacterial infections are in most cases effectively cleared by the immune system of a competent host. Some infections and other causes of inflammation such as solar exposure to the skin, prolonged tobacco smoke or chemicals, can also lead to prolonged inflammatory processes. In these chronic up-regulated situations, products of cyclooxygenase activity, or nitric oxide accumulating at the local inflammatory site lead to augmented cell proliferation and death. This is often be linked to hypermethylation of promoter regions in tumor-suppressor and/or pro-apoptotic genes. Persistence of defects in the apoptotic machinery provokes necrotic cell death and the release of cellular contents, which in turn enhances cell growth, cancer progression and infiltration of leukocytes including tumor-associated mast cells and macrophages.

Several factors, such as: 1) the nuclear protein HMGB1, 2) the S100 family of molecules; 3) purine metabolites, ATP, AMP and uric acid, and 4) heat shock proteins have emerged as relevant mediators or "endogenous damage or danger signals" to recruit inflammatory cells, to promote wound healing and associated stromagenesis, angiogenesis; and ultimately to modulate immune functions [10]. Until recently, methods to measure necrotic death in patients were not available. The application of proteomics to identify factors, such as HMGB1 in serum of cancer patients, has revealed elevated serum levels in patients with metastatic melanoma, pancreatic cancer and others [10]. The correlation of these serological markers of necrotic cell death with histological patterns, genetic resistance to apoptotic death in animal models could lead to novel targets for immune therapy, such as antibodies to HMGB1, in order to interrupt the "circolo vizioso" of this "addiction to death" which promotes tumor growth [9].

Current attempts for cancer therapy focused on vaccination to antigenic targets or application of cytokines have resulted in measurable anti-tumor reactivity in the blood; however, these therapies have mostly failed to show a correlation with tumor outcome or progression. Therefore, to more completely understand and identify factors assessing tumor death could inform and drive the development of more effective biological therapies for cancer patients. Sample acquisition in the blood includes serum/protein collection for Seldi-Tof mass spectrometry; and the collection of cells for microarray, proteomics, and high contents screening via cellomics. Protein chip Seldi-Tof MS has been already successfully used to discriminate serum expression profiles in various cancer types [11-13]. The complexity of these advanced, high-throughput technologies will exponentially increase the amount of data, with the consequence that the main activities of future biological and medical laboratories will be in data analysis and integration rather than in data collection. Therefore, specialized teams are required for collaboration efforts in order to manage data warehousing, mining and analysis, and thus establishing networks for the identification and application of biomarkers.

Beside proper study design, the models chosen to perform data classification and to estimate classification errors are highly critical for the complex data analysis. The identification of diagnostic markers for cancer, or markers to identify responders vs. non-responders to therapy requires systematical analysis of healthy vs. diseased, then of benign inflammatory disease vs. malignant cancer. Thus, methods to perform statistical analysis (e.g. permutation, randomization) are powerful, intuitive and provide an objective position from which to assess results. To handle these complex data analysis problems, the University of Pittsburgh has formed the Pittsburgh Supercomputing Center (PSC) headed by Dr. Arthur W. Wetzel, in a joint effort with Carnegie Mellon University and Westinghouse Electric Company, and is to date the most powerful open-resource computer available.

## Imaging tools and Technologies for Translational Research

There are many examples of the value of weaving molecular imaging into Investigational New Drug Development. At the same time, the scale of the initial investments required vs. perceived benefits may not gain the necessary support of decision makers for application into development programs. There is a clear need to educate on the power and limitations of nuclear imaging techniques within the context of enhancing new drug development. Within this context, a primary goal for TR is to emphasize the cultural and operational shifts required of various stakeholders including academia, in order to better partner with industry.

The term imaging covers a range of available techniques, including discovery autoradiography, small animal imaging (PET and MRI), traditional anatomical imaging (Ultrasound, MRI, CT), functional imaging (MRI, PET, SPECT) and many new tracers are available as are techniques with increased sensitivity to enable micro-doing studies (AMS) [14]. Nuclear imaging techniques are powerful tools and can be used for a number of development objectives. These include a number of goals described below.

Firstly demonstrating drug penetration into the tissue of interest and co-localization or binding with the intended target through receptor occupancy (e.g., labeled ligand displacement), including describing dose vs. target occupancy curves remains a key objective an done used frequently in early clinical research. A second objective involves the quantification of a compound's pharmacokinetic (PK) profile using radio-labeled compound, an analysis that can be performed on a region of interest basis e.g. to assess time on target as well as potential therapeutic benefits vs. side effects. Additionally, imaging can be used to quantify pharmacodynamic (PD) effects of drug action and their relationship to administered dose. In combination, PK/PD information thus derived can be used to select a dose with which to test the clinical hypothesis or help quantify the therapeutic index. From a TR perspective all these techniques can be applied in the discovery and preclinical phases to facilitate compound selection and optimization as well as in the clinical phases.

A key question emerges in applying these technologies: "How best to get it done" and the debate of internal imaging centers vs. external networks and academic relationships quickly emerges. On balance, it is clear that there is not one ideal solution here rather in general a collaborative approach between industry and academia is recommended. As a consumer of medical imaging, industry is a critical player in driving innovation and the paradigm shift towards more frequent yet appropriate utilization. However, a partnership approach ultimately generates better value and cost-effectiveness for the Imaging discipline as a whole.

## Conclusions and path forwards

TR is an approach to foster communication between the scientific community and clinical practitioners. To maximize the value this can bring requires that public and governmental education has to be improved in order to leverage understanding and advocacy. There are many benefits to be accrued from this, not least of which being for the patient that is waiting for meaningful therapeutic advances. New drugs have to be developed fast and show effect on the right target at the earliest possible stage of development in order for industry to become more innovative and productive and medicines to be less expensive.

Amongst other specific aspects required, are the strengthening of educational opportunities for physician scientists to help prepare them to conduct effective TR. At the same time, discovery science should be conducted by scientists who have been trained in relevant disciplines including cell biology and pharmacology as well as molecular biology. This in turn requires grant support for TR-related projects. Specifically, young scientific investigators should have more access to grants from governmental bodies and foundations in order to conduct research on clinical samples. This funding is largely in the hands of government leadership. Other points for disseminated education include the availability of a plethora of tools available to conduct and advance TR and development opportunities that include high quality clinical sample collection.

Lastly, since TR is information intensive, considerable efforts are required to provide accessible databases and share knowledge. To help ameliorate this gap and provide access to information derived from human experimentation and to optimize the communication between clinicians and scientist, **Dr. Marincola **founded the *Journal of Translational Medicine*, an Open Access, peer-reviewed online journal, so that more therapeutic insights may be derived from new scientific ideas – and vice versa .

In conclusion, TR represents a team effort, since no single constituency can be fluent in all aspects, and thus a concerted effort is needed amongst translational researchers to convince stakeholders and legislators of the need to support TR efforts, and thus maximize its potential.
